# Circulating cell death biomarker: good candidates of prognostic indicator for patients with hepatitis B virus related acute-on-chronic liver failure

**DOI:** 10.1038/srep14240

**Published:** 2015-09-18

**Authors:** Zhujun Cao, Fengdi Li, Xiaogang Xiang, Kehui Liu, Yuhan Liu, Weiliang Tang, Lanyi Lin, Qing Guo, Shisan Bao, Qing Xie, Hui Wang

**Affiliations:** 1 Department of Infectious Diseases, Ruijin Hospital, Shanghai Jiao Tong University School of Medicine, Ruijin Second Road 197, New Huangpu District, Shanghai, China; 2 Discipline of Pathology, School of Medical Sciences and Bosch Institute, University of Sydney, Australia

## Abstract

Investigations on survival of patients with hepatitis B virus related acute-on-chronic liver failure (HBV-ACLF) are sparse and urgently needed. The current study aimed to evaluate the prognostic value of circulating cell death biomarkers (M30-anigen, M65-antigen and HMGB1) for HBV ACLF. In this prospective study (2/2013–8/2014), 94 patients including 54 HBV-ACLF and 40 chronic hepatitis B (CHB) patients were recruited. 40 healthy controls (HC) were also recruited. HBV-ACLF were followed up for 3 months for short-term mortality. All three biomarkers were significantly elevated in HBV-ACLF compared with CHB or HC. M30- and M65-antigens could significantly discriminate between non-survivors and survivors in HBV-ACLF. However, HMGB1 showed no prognostic value. By Cox regression analysis, M30- and M65-antigens and MELD were identified as independent predictors for short-term mortality. A novel prognostic model, MELD-CD (MELD-cell death) was established based on the multivariate results. The adjusted Harrell’s C-index of MELD-CD was 0.86 (*P* < 0.001) and was significantly higher (*P *< 0.001 for all) than the currently used models, MELD (C-index, 0.71, *P *< 0.001), MELD-NA (0.67, *P *< 0.001), CTPs (0.61, *P* < 0.05). Dynamic analyses further confirmed the prognostic utility of M30- and M65-antigen. Future studies are warranted to validate the results.

Acute-on-chronic liver failure (ACLF) is a devastating syndrome involving an acute deterioration of liver function in patients with chronic liver diseases, secondary to superimposed liver injury or other factors such as infection, which leads to end-organ failure. Hepatitis B virus (HBV) has been the leading cause of ACLF in the Asia-Pacific region and the mortality has remained at over 50% during the past 20 years^1^,^2^,^3^. Clinical treatment of HBV-related ACLF (HBV-ACLF) patients is difficult owing to diverse complications and rapid progression of the disease. Liver transplantation benefits very few patients due to shortage of liver donors. Therefore, early prediction of a lethal outcome is essential for HBV-ACLF patients to receive optimal treatment.

HBV-ACLF is characterized by an acute liver injury insult, often on the basis of pre-existing chronic hepatitis B virus infection. Acute deterioration directly exaggerates liver injury and thereby leads to a mass death of hepatocytes and severe hepatic inflammation^4^. Mounting evidence supports a contributory role of hepatocyte death, apoptosis or necrosis, in the progression of ACLF^5^,^6^,^7^,^8^,^9^. It is reasonable to speculate on the important role of cell death biomarkers in evaluating liver injury and providing prognosis for HBV-ACLF patients, yet few data is available.

Studies in non-alcoholic fatty liver disease, chronic hepatitis C, CHB and drug induced liver injury have earlier reported the ability of circulating hepatocyte biomarkers such as M30-antigen, M65-antigen and high mobility group box-1 (HMGB1) in reflecting disease severity, discriminating etiologies and providing prognosis information^10^,^11^,^12^,^13^,^14^. M30-antigen is a caspase cleaved neo-epitope at Asp_396_ of Keratin-18 (K-18, a major member of the type-I intermediate filaments), and the serum level of M30-antigen indicates apoptotic hepatocyte death. M65-antigen is exposed on all intact and fragmented K-18 variants released from destroyed hepatocytes, and thus serum level of M65-antigen represents the total death of hepatocytes^15^. HMGB1 is a highly conserved, abundant, non-histone nuclear protein expressed in almost all eukaryotic cells. In response to inflammatory stimuli, HMGB1 is released from immune cells^16^. Studies have shown that HMGB1 is released from both apoptotic and necrotic cells, thus the serum level of HMGB1 is indicative of total cell death^17^,^18^.

In order to investigate the potential value of these cell death biomarkers in HBV-ACLF, we therefore measured the circulating levels of M30-antigen, M65-antigen and HMGB1 in our study population of HBV-ACLF patients, CHB patients and healthy controls. We hypothesized that these potential cell death biomarkers would be significantly elevated in HBV-ACLF patients compared with CHB and control groups. In patients with poor outcomes (death or underwent liver transplantation), the levels of these biomarkers would presumably be even higher compared with survivors. These cell death biomarkers, in combination with currently used prognosis methods such as MELD, MELD-NA or CTP would hopefully provide a more prompt, precise and powerful prognosis for HBV-ACLF patients.

## Results

### Host characteristics

In our 54 HBV-ACLF cohort, there were 47 men (87.04%) and 7 (12.96%) women with a mean age of 45.54 ± 1.45, among which 59.26% (*n* = 32) were cirrhotic. As expected, HBV-ACLF patients had significantly higher liver enzymes, total bilirubin (TB) and creatinine levels as well as lower albumin, hemoglobin levels and blood platelet counts than CHB patients. In addition, the results of our 3 months follow-up showed that 22 HBV-ACLF patients survived and 32 patients died. No patient in our study received liver transplantation. Significant differences were found between survivors and non-survivors in terms of age, aspartate aminotransferase (AST), HBV-DNA, Total bilirubin, INR (international normalized ratio), MELD, MELD-NA and CTP scores, as described in Table 1.

### Serum levels of cell death biomarkers are markedly elevated in HBV-ACLF

**Figure 1:**
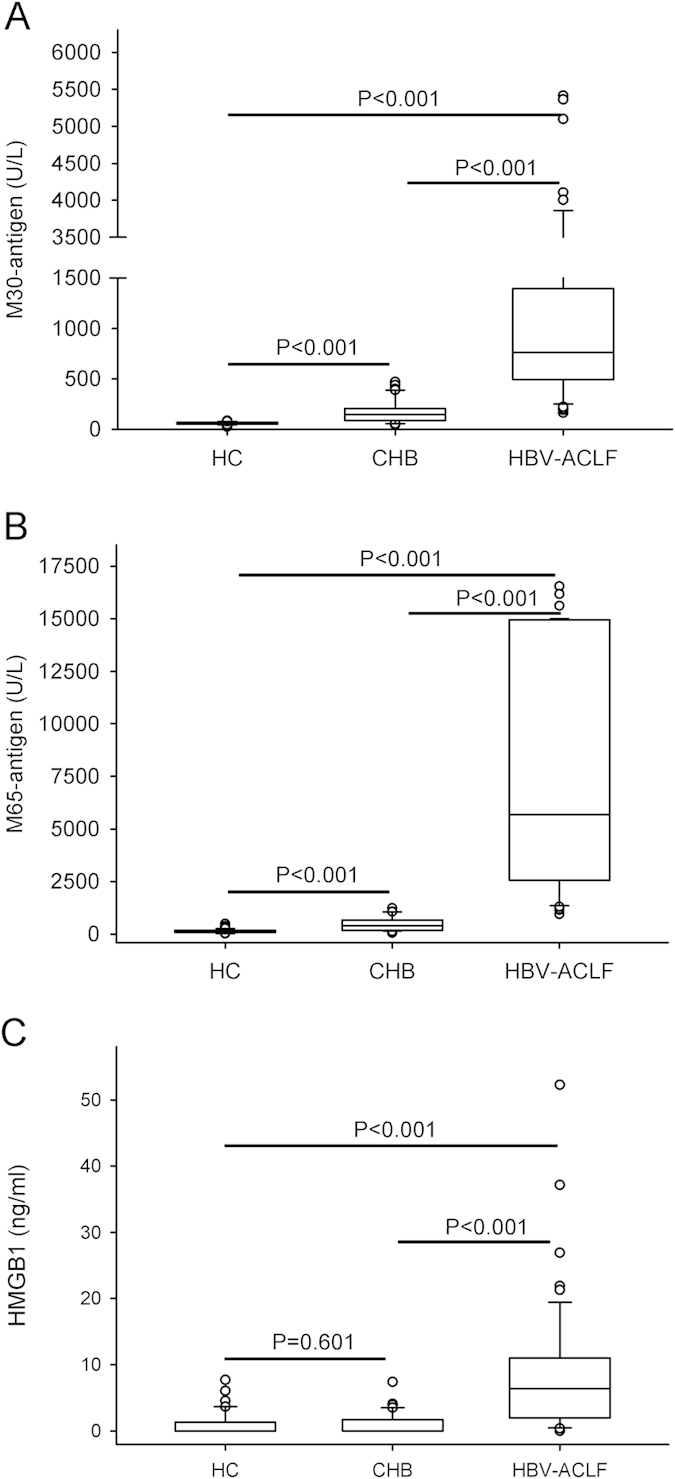
Serological detection of M30-antigen (A), M65-antigen (B) and HMGB1 (C) in healthy controls, CHB and HBV-ACLF patients. Results were presented as box plots including medians and 25th and 75th percentiles. The Kruskall-Wallis test was used for comparisons among multi-groups followed by Mann-Whitney U test for comparisons between two groups. In all three assays, cell death biomarkers were significantly elevated in HBV-ACLF patients compared with healthy controls (*P* < 0.001) and CHB patients (*P*< 0.001). In contrast to M30-antigen and M65-antigen, which discriminated (*P*< 0.001) CHB patients from healthy controls, no significant difference was found between the levels of HMGB1 (*P*= 0.601) in the two groups.

Because massive hepatocyte death has been implicated in ACLF, we analyzed the ability of different cell death biomarkers (M30-antigen, M65-antigen and HMGB1) to discriminate between HBV-ACLF and CHB patients or HC. All three biomarkers discriminated significantly (*P* < 0.001) between HBV-ACLF patients and CHB patients and either healthy control individuals (Fig. 1). Whereas seral M30- and M65-antigen from CHB patients were significantly (*P* < 0.001) higher than HC (Fig. 1A,B), no significant difference of seral HMGB1 was found between CHB patients and HC (Fig. 1C).

### Cell death biomarkers are significantly correlated with ALT, AST, TB, INR, albumin and HBV-DNA

To further investigate the clinical significance of the markedly higher levels of circulating cell death biomarkers in HBV-ACLF patients, we performed regression analyses to compare levels of cell death biomarkers with well-established liver injury markers including ALT and AST, with HBV-DNA load and additionally with albumin, TB and INR, since decreased albumin, hyperbilirubinemia and coagulopathy are the hallmarks of liver manifestation of ACLF patients. Spearman correlation coefficient was first calculated to analyze any correlation among the three measured biomarkers. Significant positive correlation was found between each two markers (between M30-antigen and M65-antigen, *r* = 0.889, *P* < 0.0001; between M30-antigen and HMGB1: *r* = 0.555, *P* < 0.001; between M65-antigen and HMGB1: *r* = 0.616, *P* < 0.001). Further regression analyses showed that all the three cell death biomarkers were significantly correlated with ALT, AST, TB and INR (*P* < 0.001). Albumin was reversely correlated with the three cell death biomarkers (*P* < 0.001 for all), supporting the data above. Moreover, M30-antigen (*r* = 0.23, *P* <0.05) and M65-antigen (*r* = 0.21, *P* <0.05) were correlated with HBV-DNA. No significant correlation was found between HMGB1 and HBV-DNA (Table 2).

### M30-antigen and M65-antigen managed to discriminate non-survivors from survivors in HBV-ACLF patients

**Figure 2:**
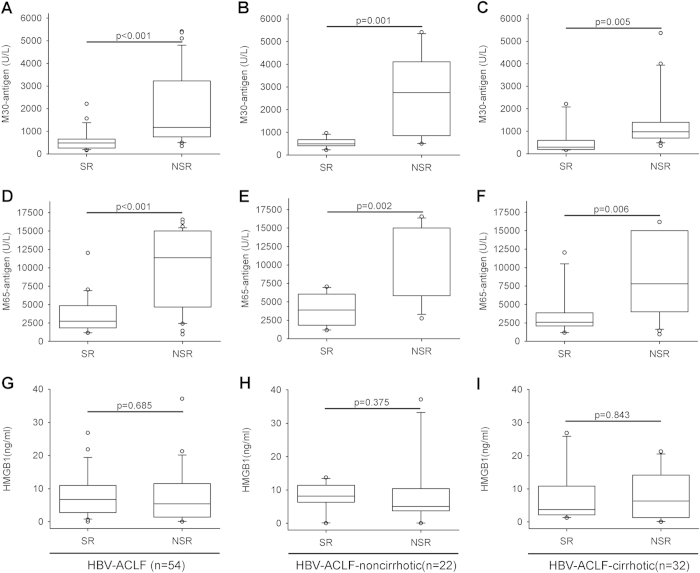
Measurements of M30-antigen, M65-antigen and HMGB1 in HBV-ACLF patients with different outcomes. Results were presented as box plots including medians and 25th and 75th percentiles. Comparisons were performed using Mann-Whitney U test between two groups. In the entire HBV-ACLF cohort, baseline serum levels of M30-antigen (**A**) and M65-antigen (**D**) in non-survivors (NSR) were significantly higher than those in survivors (SR) and no significant difference was found through HMGB1 assay (**G**). In the subgroups, non-cirrhotic HBV-ACLF patients and cirrhotic patients, baseline serum levels of M30-antigen (**B**,**C**) and M65-antigen (**E**,**F**) in non-survivors (NSR) were significantly higher than those in survivors (SR). HMGB1 did not discriminate between the two different clinical outcomes (**H**,**I**).

Based on the previous results, we came to the hypothesis that if these biomarkers would play a prognostic role. To this end, we followed up the HBV-ACLF participants for 90 days to identify the short-term outcomes. In our 54 HBV-ACLF patient cohort, 22 of them survived (SR) and 32 of them died (NSR) within the follow-up period. Baseline serum M30-antigen, M65-antigen and HMGB1 levels were measured upon the diagnosis of HBV-ACLF. As expected, baseline median seral M30-antigen from the NSR group were ~2.5 fold higher (*P* < 0.001) than those from the SR group. Likewise, baseline median seral M65-antigen from the NSR group were ~4.0 fold higher (*P* < 0.001) than those from the SR group (Fig. 2A,D). Whereas no significant difference in baseline serum HMGB1 levels was observed between SR and NSR (Fig. 2G). To adjust the effect of cirrhotic status, we evaluated the serum levels of the three biomarkers in HBV-ACLF patients by stratification on non-cirrhotic or cirrhotic HBV infection status. Significant difference of M30-antigen (*P* < 0.01) and M65-antigen (*P* < 0.01) between non-survivors and survivors were found in the cirrhotic group, as well as, in non-cirrhotic group (M30-antigen, *P* < 0.01, M65-antigen, *P* < 0.01) (Fig. 2B,C,E,F). As for HMGB1, no significant difference between survivors and non-survivors was observed in all groups (Fig. 2H,I).

### Good performance was provided by the novel prognostic model, MELD-CD in predicting the prognosis of HBV-ACLF patients

**Figure 3:**
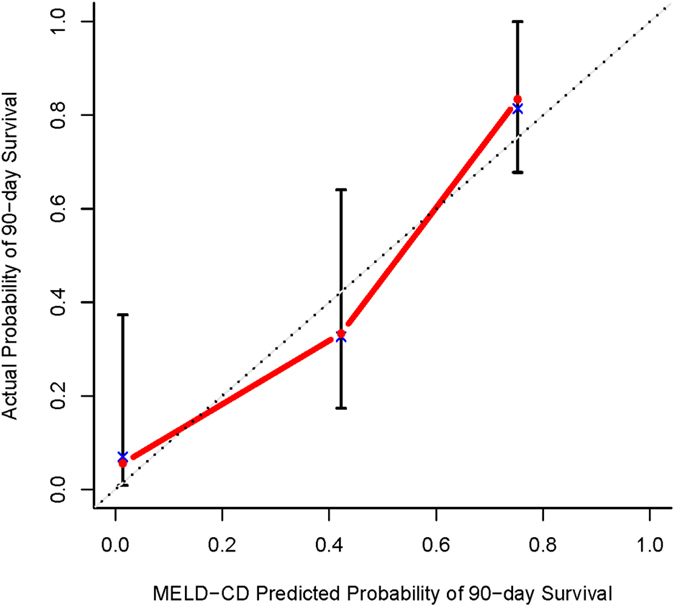
The calibration curve by bootstrap method for predicting patient survival at 90 days in the HBV-ACLF cohort. Model-predicted probability of 90-day survival is plotted on the x-axis; actual 90-day survival is plotted on the y-axis.

Since the outcome of HBV-ACLF is time to an event, univariate and multivariate Cox regression analyses were performed to identify independent predictors for prognosis in HBV-ACLF. M30-antigen, M65-antigen and MELD score were identified as three independent predictors for 90-day mortality of HBV-ACLF patients (Table 3). Consequently, a novel prognostic model, MELD-CD (MELD-cell death) was established based on the multivariate analyses. The original C-index of MELD-CD was 0.87 (95%CI, 0.82 to 0.92, *P* < 0.001) and was further corrected to 0.86 when adjusting for bias by the bootstrap method. The calibration curve demonstrated good agreement between prediction and observation in the probability of 90-day survival (Fig. 3). The corrected C-index of MELD-CD was significantly (*P*  < 0.001) higher than any of the conventional prognostic models, MELD (0.71, 95%CI, 0.62–0.80, *P* < 0.001), MELD-NA (0.67, 95%CI, 0.58–0.76, *P* < 0.001), CTPs (0.61, 95%CI, 0.51–0.70, *P* < 0.05).

### Dynamic changes of M30-antigen and M65-antigen levels in HBV-ACLF patients

To further explore the ability of hepatocyte death associated biomarkers, and the underlying mechanisms of cell death in the development and progression of HBV-ACLF, we evaluated the dynamic changes of M30-antigen and M65-antigen levels during the first 4 weeks of hospitalization in a subgroup of HBV-ACLF patients (*n* = 14), among which, 6 patients recovered and survived by the end of the 90 days follow-up period and 8 patients died, in spite of the intense medical care.

**Figure 4:**
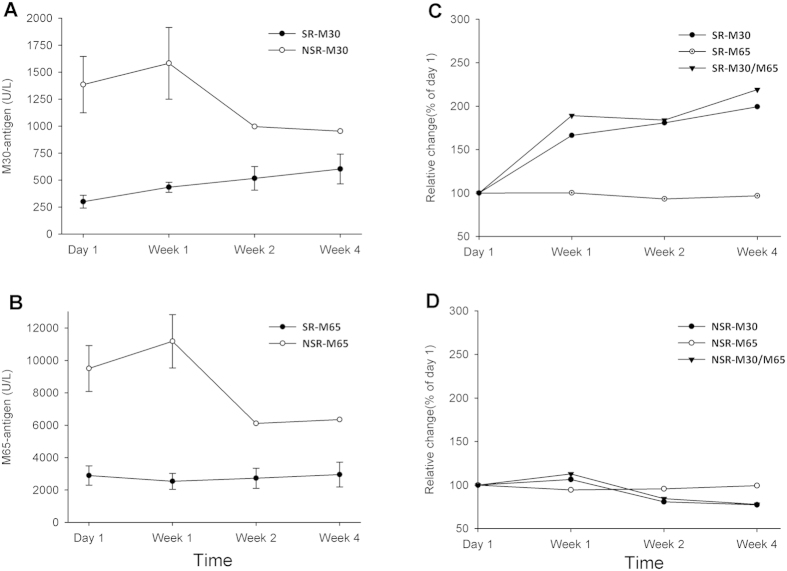
Serial measurements of M30-antigen and M65-antigen in a subset of HBV-ACLF patients. Seral M30-antigen (**A**) and M65-antigen (**B**) from 6 survivors and 8 non-survivors were measured upon diagnosis and 1, 2, 4 weeks after diagnosis. Results were presented as the mean ± SEM. Changes in M30-antigen, M65-antigen and the apoptosis rate (M30/M65) were shown as a percentage of day 1 values. The upper right panel (**C**) showed the mean changes in M30-antigen, M65-antigen and M30/M65 in the 6 survivors with HBV-ACLF, in which the M65-antigen remained relatively constant during the first 4 weeks while the M30-antigen and apoptosis rate continually increased. The lower right (**D**) panel showed a representative example of a non-survival HBV-ACLF patient with a slightly increased apoptosis rate at week 1 and a decline at week 2 and week 4.

Among this subset of 14 patients, mean serum levels of M30-antigen and M65-antigen were higher in non-survivors than survivors throughout the first 4 weeks (Fig. 4A,B). Significance analyses were performed at day 1 (M30-antigen, *P* < 0.001; M65-antigen, *P* < 0.01) and week 1 (M30-antigen, *P* < 0.01; M65-antigen, *P* < 0.01), though we failed to carry out measurements for the remaining time period since only one patient remained alive in the NSR group 10 days post admission.

Furthermore, we utilized the apoptosis marker, M30-antigen, the total hepatocyte death marker, M65-antigen, and the ratio of them, M30/M65 as the apoptosis rate to identify the characteristics of cell death modes during the progression of HBV-ACLF, Dynamic changes in apoptosis levels, indicated as a percentage of the day 1 values of M30-antigen, showed a continuous increase in all 6 survivors (Fig. 4C). However, in contrast, the total death levels remained relatively constant throughout the first 4 weeks of hospitalization, which lead to a gradual increase in the apoptosis rate (M30/M65) in survival patients. In a representative example of non-survival patients with HBV-ACLF (Fig. 4D), the change pattern in apoptosis rate was strikingly different. No obvious elevation of the apoptosis rate was found in the non-survival patient and a decrease in the apoptosis rate was found at week 2 and week 4.

## Discussion

Currently, survival studies on HBV-ACLF patients from Asia-Pacific areas are rare. Prospective collection of survival data is urgently needed to develop novel biomarkers or scoring systems to facilitate decision making on optimal treatment time and regimens such as intensive care alone, artificial liver support or liver transplantation^4^. Given the increasingly important roles for apoptotic and necrotic cell death in the pathogenesis of HBV-ACLF, we aimed to investigate three hepatocyte death-related biomarkers in HBV-ACLF patients and further evaluate the potential prognostic value of them in predicting poor prognosis.

For the measurement of hepatocyte death biomarkers in the cohort of 54 HBV-ACLF patients with 32 (59.26%) cirrhotic patients, we first identified markedly elevated levels of M30-antigen, M65-antigen and HMGB1 in HBV-ACLF patients compared with healthy controls and CHB patients. In contrast to M30- and M65-antigens which were further demonstrated to be significantly higher in CHB patients than HC, HMGB1 biomarker could not discriminate between CHB and HC. HMGB1 is more an immune responsive alarmin than a cell death biomarker^19^ while half of the CHB patients in our current study are not in the immune-active phase with moderate level of ALT. It is reasonable that low level of HMGB1 was detected from patients with chronic infection of HBV because excessive amounts of HMGB1 was reported to be released mainly in lethal systemic inflammatory diseases, e.g. severe sepsis^20^.

Significant correlations of these biomarkers with ALT, AST, TB and INR and reversely with albumin were found. ALT/AST has long been used as liver injury indicators which is also confirmed by the detection of cell death biomarkers in the current study. More importantly, it is acknowledged that acute hepatic decompensation would result in decreased albumin, hyperbilirubinemia and prolongation of the INR. Therefore, the consistency between the elevation of cell death level and the changes of TB, INR or albumin indicates the potential usefulness of cell death biomarkers in reflecting the severity of HBV-ACLF.

Moreover, we also observed a significant positive correlation between HBV-DNA and M30-antigen or M65-antigen. Actually, M30- and M65-antigens may be modified following anti-viral treatment. This is supported by the findings from other studies, showing that both M30-antigen and M65-antigen concomitantly declined with decline of HBV-DNA^21^,^22^,^23^. The kinetics of these markers from HBV-ACLF patients will be determined in our future study. Additionally, no significant correlation was found between HMGB1 and HBV-DNA in the current study. Despite the fact that HMGB1 is indicative of cell death level, circulating HMGB1 level seems to be affected by multiple signaling pathway, including ROS, Calcium, NO, TNF, Notch, MAPK and STAT pathways^24^. The precise role of HMGB1 in the development of HBV-ACLF is currently being investigated.

M30- and M65-antigens were clearly shown to be significantly elevated in non-survivors compared with survivors while serum HMGB1 was not significantly higher in non-survivors than survivors which was contrary to the results of M30- and M65-antigen. Another report has also suggested a limited role of HMGB1 in determining outcomes in acute liver failure (ALF) patients^25^. In our opinion, the release of HMGB1 into the circulation is rather complicated than we have just thought. Apart from the passive release by dead cells, active release from immune cells, fibroblasts, or epithelial cells is also a main source of circulating HMGB1.

Cell death biomarker based prognostic models are more accurate than the conventional prognostic methods for predicting prognosis in ALF patients^13^,^14^. M30-antigen based model (ALFSG index) seems to be more accurately predict ALF outcomes than MELD score^13^. M65-antigen based model (MELD-M65) is more sensitive and specific in predicting ALF in than MELD or KCC (King’s College Criteria)^14^. In the current study of HBV-ACLF, we established a novel prognostic model MELD-CD which integrated M30-antigen, M65-antigen and MELD score. We could demonstrate that the M30- and M65-antigen based model (MELD-CD) provides satisfactory predicting performance for 90-day mortality in HBV-ACLF patients and shows significantly better performance than MELD, MELD-NA and CTPs. Currently, there is no prognostic methods specifically developed for HBV-ACLF prognosis. Models like MELD are the temporary alternative method for predicting prognosis for HBV-ACLF patients. However, it is critical to evaluate quickly the severity of HBV-ACLF and subsequently determine the potential outcome to optimize treatment regime for specific patients. The newly established model, incorporating MELD and hepatocyte death biomarkers (M30- and M65-antigens), meets the criteria for outcome prediction due to the early applicability and high accuracy. Furthermore, MELD-CD emphasizes the prognostic value of cell death biomarkers which should not be neglected as well when assessing severity of HBV-ACLF. Nevertheless, it should be aware that the size of the patient cohort in the current studied was rather small with no external validation and whether MELD-CD could be applied to clinical prediction of prognosis in HBV-ACLF patient needs further validation in a larger cohort of patients, using well-designed prospective studies carried out in multi-centers.

To get a closer insight into the clinical process of HBV-ACLF and to more accurately predict the prognosis, we serially measured the apoptosis marker, M30-antigen and the marker for total hepatocyte death, M65-antigen in 14 HBV-ACLF patients, composed of 6 survivors and 8 non-survivors during the first 4 weeks of hospitalization. Both M30-antigen and M65-antigen accurately predicted the prognosis of these patients, further confirming the prognostic value of these two novel biomarkers in HBV-ACLF patients. Clearly, excessive hepatocyte death plays a fundamental role in the development and progression of ACLF. Classical modes of cell death, such as apoptosis and necrosis, and other forms of hepatic cell death including autophagy, necroptosis and pyroptosis^26^,^27^,^28^ are believed to be involved in the pathophysiology of ACLF. However, question regarding which cell death modes predominates over others or whether there is a transformation or balance between different death modes during the progression of ACLF remained unveiled.

In efforts to better characterize cell death modes in the clinical course of HBV-ACLF, we discovered a gradual increase in the apoptosis rate (M30/M65) in 6 survivors. The total cell death marker M65-antigen levels in the serum of survivors was relatively stable but the apoptotic marker M30-antigen levels in the seral of survivors kept increasing during the hospitalization, suggesting an important role of apoptosis in the recovery of HBV-ACLF, as such results were not observed in the representative of non-survivors. Similar results were also demonstrated in patients with ALF^29^. Despite the fact that a high apoptosis level contributes to strong caspase activation, caspase may participate in the regeneration processes of the liver as well, so that elevated levels of apoptosis leads to the final recovery of HBV-ACLF patients. The function of caspases in liver regeneration is best exemplified by a report showing hepatocyte proliferation attenuated by the hepatocyte-specific knockout of caspase-8^30^. Nevertheless, limited by the comparatively low numbers of HBV-ACLF patients we studied, whether caspases are indeed directly involved in recovery from HBV-ACLF definitely requires further investigation. It is also recommended that animal models be developed to further scientifically characterize ACLF and to help better define hepatocyte death mechanisms, thus permitting the use of informed targeted therapies.

In conclusion, our study not only provides information of serum cell death level in healthy volunteers, CHB patients and HBV-ACLF patients but also highlights the significance of M30-antigen and M65-antigen as prognostic biomarkers for HBV-ACLF patients. The novel prognostic model, MELD-CD was demonstrated to provide significantly better performance than MELD, MELD-NA and CTP scores in predicting 90-day mortality for HBV-ACLF patients. Time series analyses further confirm the utility of M30- and M65-atnigen in provided prognostic information. A gradual increase in the apoptosis rate (M30/M65) was demonstrated within the clinical course of survivors, which suggest an underlying role of apoptosis in recovery from HBV-ACLF. Future studies are warranted to validate the results and further explore the underlying mechanism.

## Methods

### Study subjects

94 patients (79.79% male, mean age 44.12 ± 1.14 years) including 40 CHB patients and 54 HBV ACLF and 40 healthy controls (45% male, mean age 31.88 ± 1.92 years) were enrolled from February 2013 to August 2014 in the Department of Infectious Diseases, Ruijin Hospital. All HBV-ACLF patients were followed up for at least 3 months to identify the status of short-term clinical outcomes, which was categorized as survival or non-survival (death or underwent liver transplantation). The start date of the follow-up was the date of the diagnosis of HBV-ACLF. Chronic HBV infection was defined as a positive hepatitis B surface antigen (HBsAg) for at least 6 months prior to the beginning of this study^31^.

Diagnosis for HBV-ACLF patients was based on consensus recommendations of the Asia-Pacific Association for The Study of the Liver (APASL)^32^. Inclusion criteria were: (1) Clear acute hepatic insult manifesting as jaundice and coagulopathy, complicated within 4 weeks by ascites and/or encephalopathy, in a patient with previously diagnosed or undiagnosed chronic liver disease. (2) Total bilirubin ≥ 5 mg/dL (85 μmol/L). (3) Prothrombin activity ≤ 40% or INR ≥ 1.5. (4) The presence of ascites and/or encephalopathy as determined by physical examination. APASL definition was chosen because it is more suitable for the Asians, particularly for Chinese ACLF patients, mainly CHB patients. Whereas diagnostic criteria proposed by EASL-CLIF Consortium^33^ is mainly based on patients with alcoholic cirrhosis, and German ALF definition is more suitable for acute liver failure^34^.

Patients were excluded if they had any of the following: (1) Evidence of hepatocellular carcinoma (suspicious foci on hepatic ultrasonography at screening or a rising serum level of α-fetoprotein). (2) Presented with other viral hepatitis or superimposed with hepatitis C virus, hepatitis D virus, hepatitis E virus, human immunodeficiency virus, non-alcoholic fatty liver disease, Wilson’s disease, metabolic liver disease, hemochromatosis, autoimmune hepatitis, primary biliary cirrhosis, primary sclerosing cholangitis or alcohol abuse.

This study complies with the declaration of Helsinki, and the study protocol was approved by the Ethics Committee of Ruijin Hospital. Written informed consent was signed by all participants or their next of kin (for patients with encephalopathy).

### Blood samples and laboratory data collection

Blood samples from 40 CHB patients and 40 healthy controls were collected at enrollment and were collected from 54 patients once they were diagnosed as HBV-ACLF. (Blood samples from 14 HBV-ACLF patients were further collected at the time point of 7, 14, 28, days post diagnosis for dynamic analysis). Blood sera were separated and stored at −20 °C within 2 hours of collection until testing. Demographic data and standard hematological, biochemical, virology and coagulation parameters were recorded.

### Serum biomarker assays

Serum M30-antigen level was quantified using M30-Apoptosenses ELISA (Peviva AB, Bromma, Sweden) that specially recognized the caspase-cleaved neo-epitope of K-18 released from apoptotic hepatocyte. The working range of the kit is 0–1000 U/L. Serum M65-antigen level was quantified using the M65 EpiDeath^®^ ELISA (Peviva AB, Bromma, Sweden) that measured both full-length K-18 and fragmented Keratin-18 (total hepatocyte death). The working range of the kit is 0–5000 U/L. Serum HMGB1 concentration was determined using HMGB1 ELISA kit (Shino-Test Corp, Oonodai, Kanagawa, Japan). The working range of this ELISA kit is 2.5–80 ng/mL. Samples presenting higher measurements than the range of the standard curve were diluted to yield satisfactory linearity.

### Prognostic models

MELD: In order to calculate the individual MELD score we used the formula as published^35^: MELD = 9.57 × Ln_Creatinine_[mg/dL] + 3.78 × Ln_Total bilirubin_[mg/dL] + 11.2 × Ln_INR_ + 6.43 × etiology (0, if cholestatic or alcoholic; 1, otherwise). MELD-NA: MELD-NA score was calculated according to the following formula^36^: MELD-NA = MELD + 1.59 × (135-Serum sodium [mmol/L]). Child-Turcotte-Pugh (CTP): The CTP classification was assessed according to standard criteria^37^.

### Statistical analyses

Data are presented as the mean ± SEM (standard error of mean), medians (25th, 75th percentile) for continuous factors or counts and percentages for categorical variables, unless stated otherwise. The Shapiro-Wilk test was used for normality analysis. For normally distributed data, comparisons between two groups were performed using independent-sample *t*-test; comparisons among multi-groups were performed using one-way ANOVA followed by Student-Newman-Keuls’test (SNK-test) for comparisons between each two groups. For abnormally distributed data, comparisons between two groups were performed using Mann-Whitney U test. The Kruskall-Wallis test was used for comparisons among multi-groups. Chi-square tests were used for the comparisons of categorical factors. Correlations between variables were analyzed by Spearman’s correlation.

Univariate and Multivariate Cox regression were performed to identify independent risk factors for poor prognosis of HBV-ACLF. Forward stepwise selection method was used for selecting variables with a predefined criteria of *P* < 0.2 for selection and *P* < 0.05 for elimination. The performance of prognostic models were measured by Harrell’s concordance index (C-index)^38^. The larger the C-index, the more accurate was the prognostic prediction. Bootstrap with 1,000 resamples were used for adjusting bias and comparing model-predicted versus observed survival probability.

All statistical analyses were carried out using SAS Ver. 9.3 (SAS Institute Inc, Cary, NC), R 3.2.0 (http://www.r-project.org/) and Sigmaplot 12.5 for Windows (Systat Software, Inc., San Jose, CA, USA). A two-tailed P < 0.05 was considered to be statistically significant.

## Additional Information

**How to cite this article**: Cao, Z. *et al.* Circulating cell death biomarker: good candidates of prognostic indicator for patients with hepatitis B virus related acute-on-chronic liver failure. *Sci. Rep.* **5**, 14240; doi: 10.1038/srep14240 (2015).

## Figures and Tables

**Table 1:** Demographic and clinical characteristics of study subjects.

	HC (*n* = 40)	CHB (*n* = 40)	ACLF (*n* = 54)			
			Total (*n* = 54)	SR (*n* = 22)	NSR (*n* = 32)	P*
Male n (%)	18 (45.00%)	28 (70.00%)	47 (87.04%)	21 (95.45%)	26 (81.25%)	0.127
Age (years)	31.88 ± 1.92	42.2 ± 1.82	45.54 ± 1.45	40.00 ± 2.29	49.34 ± 1.57	0.001
BMI (kg/m^2^)	21.90 ± 0.42	22.61 ± 0.47	24.01 ± 0.43	23.63 ± 0.57	24.28 ± 0.62	0.469
HBeAg positive n (%)	/	22 (55.00%)	22 (40.74%)	12 (54.54%)	10 (31.25%)	0.087
Cirrhotic n (%)	/	/	32 (59.26%)	11 (50.00%)	21 (65.63%)	0.251
ALT (IU/L)	12.73 ± 0.53	96.53 ± 16.75	222.09 ± 39.96	197.09 ± 67.44	239.28 ± 49.65	0.081
AST (IU/L)	17.68 ± 0.84	53.88 ± 7.13	214.19 ± 24.55	141.95 ± 19.51	263.84 ± 36.97	0.012
albumin (g/L)	43.63 ± 0.51	37.75 ± 5.45	28.76 ± 0.61	28.77 ± 1.08	28.75 ± 4.05	0.985
BPC ( × 10^9^)	/	157.67 ± 9.78	84.28 ± 5.47	92.09 ± 9.62	78.91 ± 6.40	0.231
hemoglobin (g/L)	/	138.41 ± 2.53	112.04 ± 3.13	109.14 ± 4.97	114.03 ± 28.07	0.448
Log_10_HBV-DNA (IU/mL)	/	4.13 ± 0.26	4.50 ± 0.24	3.74 ± 1.42	5.02 ± 0.31	0.007
TB (μmol/L)	12.73 ± 0.82	37.40 ± 10.46	447.03 ± 24.68	368.13 ± 31.33	501.28 ± 32.67	0.007
Cr (μmol/L)	/	74.69 ± 8.45	98.11 ± 6.77	86.50 ± 5.23	106.09 ± 10.70	0.398
INR	/	/	2.09 ± 0.08	1.81 ± 0.07	2.27 ± 0.11	0.002
MELD score	/	/	28.09 ± 0.71	24.95 ± 0.67	30.25 ± 0.95	< 0.001
MELD-NA score	/	/	32.77 ± 1.32	27.92 ± 1.27	36.11 ± 1.83	< 0.001
CTP score	/	/	11.65 ± 0.24	10.74 ± 0.33	12.25 ± 0.28	0.001

Data were presented as numbers (percentage) and mean ± SEM.

**Abbreviation:** BPC, blood platelet counts; TB, total bilirubin; Cr, Creatinine; INR, international normalized ratio; HBeAg, hepatitis B e antigen.

Mann-Whitney U test and independent-samples *t*-test for continuous factors and Pearson’s chi-square test for categorical factors were used as appropriate.

*P values correspond to the comparison of the SR and NSR groups of HBV-ACLF patients.

**Table 2:** Correlations between cell death biomarkers and ALT, AST, TB, INR, albumin or HBV-DNA load in study subjects.

	M30-antigen (U/L)		M65-antigen (U/L)		HMGB1 (ng/mL)	
	*r*	*P*	*r*	*P*	*r*	*P*
M65-antigen (U/L)^#^	0.89	<0.001				
HMGB1 (ng/mL)^#^	0.56	<0.001	0.62	<0.001		
ALT (IU/L)^#^	0.81	<0.001	0.77	<0.001	0.48	<0.001
AST (IU/L)^#^	0.89	<0.001	0.87	<0.001	0.53	<0.001
TB (μmol/L)^#^	0.78	<0.001	0.80	<0.001	0.52	<0.001
INR*	0.76	<0.001	0.79	<0.001	0.52	<0.001
Albumin (g/L)^#^	−0.76	<0.001	−0.75	<0.001	−0.44	<0.001
Log_10_ HBV-DNA(IU/mL)*	0.23	0.023	0.21	0.047	0.06	0.598

^#^Spearman correlation analyses between cell death biomarkers and ALT, AST, TB or albumin were performed in all study subjects (*n* = 134).

*Spearman correlation analyses between cell death biomarkers and INR or Log_10_ HBV-DNA were performed in CHB and HBV-ACLF patients (*n* = 94).

**Table 3:** Univariate and multivariate analyses of the HBV-ACLF cohort.

	Univariate analysis			Multivariate analysis^#^		
	*P*	HR	95%CI	*P*	HR	95%CI
Gender (male vs female)	0.283	0.614	0.252–1.495			
Age (years)	0.005	1.048	1.014–1.083			
BMI (kg/m^2^)	0.371	1.056	0.937–1.191			
ALT (IU/L)	0.261	1.001	1.000–1.002			
AST (IU/L)	<0.001	1.004	1.002–1.006			
WBC (×10^9^/L)	<0.001	1.109	1.059–1.161			
hemoglobin (g/L)	0.140	1.012	0.996–1.028			
albumin (g/L)	0.898	1.005	0.931–1.085			
BPC (×10^9^/L)	0.322	0.995	0.986–1.005			
TB (μmol/L)	0.002	1.003	1.001–1.005			
Cr (μmol/L)	0.053	1.007	1.000–1.014			
INR	0.001	2.516	1.430–4.425			
Sodium (mmol/L)	0.146	0.952	0.891–1.017			
HBeAg positive (yes vs no)	0.035	0.446	0.211–0.946			
Log_10_HBV-DNA (IU/mL)	0.002	1.336	1.108–1.612			
M30-antigen (U/L)*	<0.001	3.864	2.408–6.200	0.044	2.027	1.683–2.371
M65-antigen (U/L)*	<0.001	4.910	2.662–9.057	0.039	2.431	2.008–2.854
HMGB1 (ng/mL)	0.464	1.013	0.978–1.049			
MELD	<0.001	1.137	1.070–1.209	0.0016	1.118	1.083–1.153
CTPs	0.012	1.311	1.060–1.622			

**Abbreviation:** WBC, white blood cell, BPC, blood platelet counts; TB, total bilirubin; Cr, Creatinine; INR, international normalized ratio; HBeAg, hepatitis B e antigen.

^#^Variables identified as *P* < 0.05 in univariate analyses were further analyzed in multivariate analysis.

*Log_e_ transformation.
